# Responses of Nitrogen-Cycling Microorganisms to Dazomet Fumigation

**DOI:** 10.3389/fmicb.2018.02529

**Published:** 2018-10-23

**Authors:** Wensheng Fang, Dongdong Yan, Xianli Wang, Bin Huang, Xiaoning Wang, Jie Liu, Xiaoman Liu, Yuan Li, Canbin Ouyang, Qiuxia Wang, Aocheng Cao

**Affiliations:** Institute of Plant Protection, Chinese Academy of Agricultural Sciences, Beijing, China

**Keywords:** soil fumigation, dazomet, N_2_O, nitrogen transfer, functional gene abundance, bacterial community composition

## Abstract

The influence of soil fumigation on microorganisms involved in transforming nitrogen remains little understood, despite the use of fumigants for many decades to control soil-borne pathogens and plant-parasitic nematodes. We used real-time PCR (quantitative polymerase chain reaction) and 16S rRNA gene amplicon sequencing techniques to monitor changes in the diversity and community structure of microorganisms associated with nitrogen transfer after the soil was fumigated with dazomet (DZ). We also examined nitrous oxide (N_2_O) emissions from these microorganisms present in fumigated fluvo-aquic soil and lateritic red soil. Fumigation with DZ significantly reduced the abundance of 16S rRNA and nitrogen cycling functional genes (*nifH*, AOA *amoA*, AOB *amoA, nxrB, narG, napA, nirK, nirS, cnorB, qnorB*, and *nosZ*). At the same time, N_2_O production rates increased between 9.9 and 30 times after fumigation. N_2_O emissions were significantly correlated with ${\mathrm{NH}}_{4}^{+}$, dissolved amino acids and microbial biomass nitrogen, but uncorrelated with functional gene abundance. Diversity indices showed that DZ temporarily stimulated bacterial diversity as well as caused a significant change in bacterial community composition. For example, DZ significantly decreased populations of N_2_-fixing bacteria *Mesorhizobium* and *Paenibacillus*, nitrifiers *Nitrosomonas*, and the denitrifiers *Bacillus, Pseudomonas*, and *Paracoccus.* The soil microbial community had the ability to recover to similar population levels recorded in unfumigated soils when the inhibitory effects of DZ fumigation were no longer evident. The microbial recovery rate, however, depended on the physicochemical properties of the soil. These results provided useful information for environmental safety assessments of DZ in China, for improving our understanding of the N-cycling pathways in fumigated soils, and for determining the potential responses of different N-cycling groups after fumigation.

## Introduction

Soil fumigation enables the production of crops of acceptable yield and quality by effectively controlling soil-borne pathogens, nematodes and weeds. Generally, root crops such as carrots, potato and ginger that have been grown in fumigated soil have fewer rejections for blemishes at harvest and better marketable yield than when they are grown in unfumigated soil (Jonathan et al., 2005; Qiao et al., 2011, 2012; Mao et al., 2014). Dazomet (DZ) is a highly effective microbicide that kills pests by a carbonylation reaction with nucleophilic sites (such as amino, hydroxyl, thiol) that may be present in enzyme molecules (Lin et al., 2000). Fumigation of soil with DZ prior to planting provides effective control of soil-borne pathogens and weeds in the production of high-value crops. It has become a valuable replacement for methyl bromide, which was phased out globally because of its harmful contribution to stratospheric ozone (Martin, 2003).

Fumigants in general kill not only target pathogens but also off-target microorganisms that can be important in the soil microbial community. Microorganisms, and in particular bacteria, are reported to significantly influence the soil quality and crop yield by regulating several important soil processes, such as organic matter decomposition, organic pollutant degradation and nutrient transformation (Álvarez-Martín et al., 2016; Crouzet et al., 2016). Previous studies reported that the fumigants chloropicrin (CP), 1,3-dichloropropene and metam sodium (MS) significantly decreased soil bacterial diversity and significantly changed the predominance of bacterial phyla in microbial communities (Liu et al., 2015; Li et al., 2017a,b; Zhang et al., 2017). Recent studies also showed that CP and MS caused significant changes in the population size and community composition of bacteria involved in N-cycling (Li et al., 2017a,b). These changes significantly affected nitrogen transforming activities, including nitrogen mineralization (Shen et al., 1984) and nitrogen reduction (Yan et al., 2013, 2017). Furthermore, previous research reported that nitrous oxide (N_2_O) production increased by 12.6 times following CP fumigation when these fumigants were combined with chemical inhibitors such as acetylene, antibacterial and antifungal agents and oxygen (Spokas et al., 2005, 2006). However, the microbial community’s role of increasing N_2_O emissions after soil fumigation was not fully investigated in these studies.

Our study examined the response of different groups of N-cycling microbes present in Jiangxi lateritic red and Beijing fluvo-aquic soils fumigated with DZ. Over a period of 59 days after fumigation, we quantified N_2_O emissions, changes to the geochemical parameters ${\mathrm{NO}}_{3}^{–}$ and ${\mathrm{NH}}_{4}^{+}$, as well as changes to the microbial biomass nitrogen (MBN) and dissolved amino acids (DAA). We also used real-time PCR to monitor the abundance of key functional marker genes (*nifH*, AOA *amoA*, AOB *amoA, nxrB, narG, napA, nirK, nirS, cnorB, qnorB*, and *nosZ*) involved in microbial N_2_-fixation, nitrification and denitrification. At the same time, we used 16S rRNA gene amplicon sequencing techniques to determine the bacterial diversity and community structure of microorganisms involved in nitrogen transformation in Beijing soil fumigated with DZ. We hypothesized that (i) DZ fumigation would produce a significant change in the abundance and community composition of nitrogen-transforming bacteria*;* (ii) N_2_O emissions would be significantly correlated with the abundance of N-cycling functional genes; and (iii) DZ fumigation would initially decrease N-cycling bacteria in the soil because of its unselective toxic effect, but this decrease would be followed shortly afterwards by an increase in these bacteria as the concentration of DZ in the soil reduced over time. The information obtained in this study will provide further insights into the N-cycling pathways in fumigated soils and into the potential responses of different N-cycling groups after fumigation.

## Materials and Methods

### Soil Samples Collection

Soil samples were taken from the top 20 cm of two agricultural fields in Beijing Shunyi (40°03′17.62″ N, 116°56′23.71″ E) and Jiangxi Yudu (26°06′31.84″ N, 115°33′51.83″ E) districts. Specifically, spade-squares (10 cm × 10 cm to a depth of 20 cm) of soil were taken from five locations along a ‘W’ line. These two samples were typical alkaline fluvo-aquic soils (‘Beijing soil’) and acidic lateritic red soils (‘Jiangxi soil’). The taxonomic names of the soils were determined according to PRC 1:1,000,000 scale soil map that was provided by the Data Center for Resources and Environmental Sciences, Chinese Academy of Sciences. Soil texture was determined using a Laser Particle Size Analyzer (Mastersizer 2000, China). Soil pH was measured when the soil: water ratio was 1:5. Salinity was measured by conductometric analysis. The physicochemical features of these soils are summarized in Table 1. The soils were pre-incubated for 7 days at room temperature (25 ± 5°C) in the dark and then adjusted to 45% water holding capacity (WHC) before fumigation.

**Table 1:** Physical and chemical properties of Beijing and Jiangxi soils.

Soil name	Sand^a^ %	Clay %	Silt %	Soil Type^b^	pH^c^	Salinity^d^ μs/cm	OM^e^ g/kg	${\mathrm{NH}}_{4}^{+}$-N^f^mg/kg	${\mathrm{NO}}_{3}^{–}$-N^f^ mg/kg
Beijing soil	72.9	2.7	24.3	Fluvo-aquic soil	7.2	899	30.7	9.4	58.9
Jiangxi soil	51.1	6.1	42.7	Lateritic red soil	4.3	136	24.5	30.4	28.1

*^*a*^Soil texture was determined using a Laser Particle Size Analyzer; ^*b*^soil taxonomic name was determined according to PRC 1:1,000,000 scale soil map. Data were provided by the Data Center for Resources and Environmental Sciences, Chinese Academy of Sciences; ^*c*^Soil pH was measured when the soil: water ratio was 1:5; ^*d*^salinity was measured by conductometric analysis; ^*e*^organic matter was measured by the potassium dichromate method; ^*f*^mineral nitrogen was extracted using 2 M KCl and measured with a continuous flow automated analyzer*.

### Experimental Setup

The soils were sieved through a 2 mm screen before any treatments were applied. Microcosms were prepared with 220 g (dry weight) of sieved soil in 500 mL Duran^®^ wide-neck glass bottles (Schott AG, Mainz, Germany). The fumigant DZ (98.5% purity) was obtained from Zhejiang Haizheng Chemical Co. Ltd., China. DZ was added at the typical field application rate of 58 mg kg^-1^ to both soil types, which were considered as treatment groups. The soil-fumigant mixture was homogenized using a spatula. The control group was treated without fumigant. Each treatment contained three replicates. Each microcosm bottle that contained soil was sealed with a butyl rubber stopper and an outlet port to allow sampling of the internal atmosphere using a syringe. The bottles were incubated at an ambient temperature of 28°C in daylight.

### Soil Sampling and Geochemical Analyses

The N_2_O emissions were measured using methods previously described (Harter et al., 2014). During the incubation stage, the soil samples in the bottles were thoroughly stirred for 10–15 min every day to create an aerobic incubation environment. At the designated time, a 10 mL gas sample was withdrawn from each bottle using a gas-tight syringe. The gas samples were transferred to a 21 mL headspace vial that was flushed with helium and evacuated (10 mL) before use. An Agilent 7890A gas chromatograph with an electron capture detector (^63^Ni-ECD) that was connected to an Agilent 7694E headspace sampler (Agilent Technologies, United States) were used to quantify the N_2_O concentrations. The gas chromatograph conditions and gas emission calculations used methods previously described (Loftfield et al., 1997; Wang and Wang, 2003). After each gas sample, the microcosm bottles were opened in a ventilation hood to vent any residual fumigant. At the designated time, 10 g soil sample was withdrawn from each bottle for geochemical and molecular biological analyses. After sampling, all bottles were pumped with fresh air and then returned to the incubator. During incubation the water content was controlled gravimetrically and adjusted each week to the initial WHC by adding deionized water from a spray bottle. Gas samples were collected every week (on days 10, 17, 24, 31, 38, 45, 52, and 59) and soil samples were collected on days 10, 24, 38, and 59. Soil samples were extracted for mineral nitrogen (${\mathrm{NH}}_{4}^{+}$-N and ${\mathrm{NO}}_{3}^{–}$-N) using 2 M KCl. The concentration of mineral nitrogen in each sample was determined using a continuous flow automated analyzer (Futura Continuous Flow Analytical System, Alliance Instruments, France). MBN was estimated using the chloroform fumigation method (Brookes et al., 1985). DAA was measured using the ninhydrin reaction method (Joergensen and Brookes, 1990).

### Microbial DNA Extraction and Real-Time Quantitative PCR

Soil samples were stored at -80°C pending DNA extraction. Soil total genomic DNA was extracted from 0.25 g of each soil sample using a MoBio Powersoil^®^ DNA Isolation Kit (Mo Bio Laboratories, United States) according to the manufacturer’s protocol. The quality and concentration of extracted DNA were determined using gel electrophoresis (1% agarose) and a NanoDrop^®^ 1000 spectrophotometer (Thermo Fisher Scientific, United States). The *nifH* gene copy number was used to quantify the abundance of bacteria capable of fixing nitrogen. The abundance of archaeal and bacterial ammonia monooxygenase and nitrite oxidases were assessed via the functional gene abundance of archaeal *amoA* (AOA *amoA*), bacterial amoA (AOB *amoA*) and *nxrB*, respectively. The abundance of bacteria capable of nitrate reduction, nitrite reduction, nitric oxide reduction and nitrous oxide reduction in denitrification were quantified by determining the copy numbers of *napA* and *narG, nirS* and *nirK, qnorB* and *cnorB*, and *nosZ* genes in the soil, respectively. Quantification of these functional marker genes as well as 16S rRNA (bacteria) was carried out using the SsoFast^TM^ EvaGreen^®^ Supermix (Bio-Rad Laboratories, United States) and gene-specific primers. Ten-fold serially diluted plasmids containing the target gene were constructed to generate a standard curve. Quantitative PCR was performed using CFX96 real-time PCR system (Bio-Rad, United States). Reactions mixtures of 20 μL contained 10 μL of 2 × SsoFast^TM^ EvaGreen^®^ Supermix, 20 ng of genomic DNA template and 0.5 μM of each primer. The details of gene-specific qPCR primers, reaction mixtures and thermal programs are listed in Supplementary Tables S1–S3. The MIQE guidelines (Bustin et al., 2009) were followed during the entire qPCR process for both evaluation and data analyses. These methods obtained a target genes amplification efficiency of 83–105%. The *R*^2^ values ranged from 0.994 to 0.999. Special amplification of target genes was confirmed by melting curve analysis, which always resulted in a single peak.

### High-Throughput Sequencing and Bioinformatics Analysis

MiSeq sequencing of the 16S rRNA genes in the V3–V4 regions using the total DNA extracted from Beijing soil microorganisms was conducted using the universal primer 338F [5′-ACTCCTACGGGAGGCAGCAG-3′] and 806R [5′-GGACTA CHVGGGTWTCTAAT-3′]. MiSeq sequencing was completed in equimolar and paired-end sequenced (2 × 300) on an Illumina^®^ MiSeq sequencer (Illumina, United States) by Majorbio Bio-pharm Technology Co. Ltd. (Shanghai, China). The sequencing data, including reads merged and filtered, were carried out using the fast length adjustment of short reads (FLASh, Johns Hopkins University, United States) and Trimmomatic (AG Usadel, Germany). Sequences < 50 bp in length with an average quality score < 20 and those with ambiguous calls were discarded for the subsequent analyses. A total of 2,681,891 high-quality 16S rRNA reads was obtained. Sequences of the 16S rRNA gene were clustered into operational taxonomic units (OTUs) at the 97% similarity level using UPARSE (version 7.1^1^). The RDP Classifier Algorithm^2^ was used to compare the taxonomy of each 16S rRNA gene sequence with the gene sequences stored in the Silva (SSU123) 16S rRNA database, based on a confidence threshold of 70%. The raw reads were deposited into the NCBI Sequence Read Archive (SRA) database (No. SRP124701). All the certified nitrogen cycle related functional microbes were collected from the NCBI database^3^ (showed in Supplementary Tables S4, S5).

### Statistical Analyses

A univariate analysis of variance (ANOVA) with the ‘least significant difference (LSD)’ test was applied using the SPSS statistics software package, version 18.0 (IBM, United States) in order to identify the major effects of fumigation on the biochemical parameters and abundance of functional genes involved in N-cycling. The univariate ANOVA was used to reveal differences between the control and the fumigated soil microcosms. All concentration or gene copy number values from the control at each time point of sampling were individually compared with the fumigation soil microcosms. The α-diversity indices Chao1, Ace, Shannon and Simpson were calculated using the bioinformatics tool Mothur to determine the diversity of the bacterial communities in Beijing soil (Schloss et al., 2009).

Hierarchical clustering and a Heat Map analyses were used to determine changes in the relative abundance of genera of bacteria involved in N-cycling. The Bray–Curtis algorithm was used to show the relative abundance of these genera according to hierarchical clustering. A Heat Map figure and statistical correlations were generated to produce a visual display of the sequencing results using heatmap-package and vegan package in R, respectively (Version 2.15.3). Spearman’s rank correlation coefficient was used to determine the relationship between N_2_O emission rate, physicochemical parameters and microbial functional genes.

## Results

### N_2_O Production Rate

**FIGURE 1:**
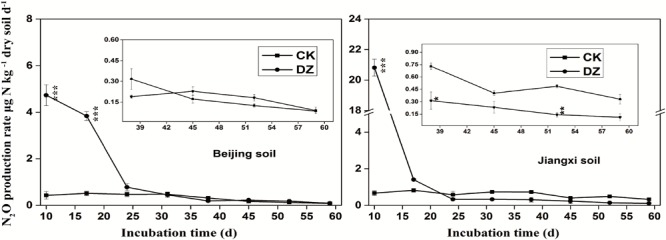
Dynamics of N_2_O production rate (μg N kg^-1^ dry soil d^-1^) following Dazomet (DZ) fumigation of Beijing soil **(left)** and Jiangxi soil **(right)**. The small inserted graphs show a magnified view of the data for the last 23 days. Error bars represent standard errors of the means (*n* = 3). Statistically significant differences between the control (CK) and DZ fumigation treatments at specific time points are indicated by asterisks according to the level of significance (^∗^*p* < 0.05, ^∗∗^*p* < 0.01, ^∗∗∗^*p* < 0.001).

N_2_O maximum production rates increased 9.9 times and 30 times within 10 days of DZ fumigation of Beijing soil and Jiangxi soil, respectively, compared with unfumigated soils (Figure 1). N_2_O emission rates rapidly decreased after DZ fumigation. They returned to emission levels similar to the control by days 17 and 24 in fumigated Jiangxi soil and Beijing soil, respectively. However, significantly lower N_2_O emissions in Jiangxi-fumigated soil were observed on days 38 (0.31 ± 0.12 vs. 0.73 ± 0.04 μg N kg^-1^ dry soil d^-1^) and 52 (0.14 ± 0.03 vs. 0.48 ± 0.02 μg N kg^-1^ dry soil d^-1^), compared with unfumigated soil.

### Changes in Physiochemical Parameters

In general, compared with the unfumigated soils, fumigation with DZ significantly increased the concentration of ${\mathrm{NH}}_{4}^{+}$-N and DAA in both soil types, whereas ${\mathrm{NO}}_{3}^{–}$-N and MBN were significantly decreased (Table 2). All these parameters recovered to levels similar to the control 38–59 days after fumigation (except ${\mathrm{NH}}_{4}^{+}$-N and ${\mathrm{NO}}_{3}^{–}$-N in Jiangxi soil) (Table 2). However, compared to Beijing soil, DZ produced larger changes in ${\mathrm{NH}}_{4}^{+}$-N and DAA concentrations that also persisted for longer in Jiangxi soil. For example, there were no significant differences in ${\mathrm{NH}}_{4}^{+}$-N and ${\mathrm{NO}}_{3}^{–}$-N concentrations between the fumigated and unfumigated Beijing soil 24 days after DZ fumigation. In contrast, there was a significantly lower ${\mathrm{NO}}_{3}^{–}$-N concentration and significantly higher ${\mathrm{NH}}_{4}^{+}$-N concentration in the fumigated Jiangxi soil compared to the unfumigated soil throughout the entire incubation period.

**Table 2:** Values of selected biochemical parameters in fumigated and unfumigated soil over time.

Soil	Parameter	Treatment	Days after fumigation (*d*)				
			0	10	24	38	59
Beijing soil	${\mathrm{NH}}_{4}^{+}$-N	CK	8.22 ± 0.91	7.21 ± 0.44	6.41 ± 1.07	5.45 ± 0.54	4.97 ± 0.69
		DZ	8.51 ± 1.51	12.4 ± 2.51^∗^	7.38 ± 0.56	5.72 ± 0.56	5.62 ± 0.23
	${\mathrm{NO}}_{3}^{–}$-N	CK	47.4 ± 1.87	49.2 ± 1.67	50.3 ± 3.56	58.6 ± 1.05	68.2 ± 1.64
		DZ	50.8 ± 2.11	42.8 ± 1.58^∗∗∗^	46.0 ± 0.55^∗^	55.5 ± 2.55	67.4 ± 8.21
	MBN	CK	29.8 ± 1.77	27.2 ± 0.54	22.9 ± 1.07	20.4 ± 0.54	19.9 ± 0.69
		DZ	29.5 ± 2.11	12.4 ± 2.51^∗∗∗^	10.3 ± 0.56^∗∗∗^	16.7 ± 1.56	17.6 ± 0.23
	DAA	CK	6.22 ± 1.76	4.23 ± 0.67	5.32 ± 0.56	5.61 ± 0.65	6.21 ± 1.64
		DZ	5.34 ± 0.88	39.8 ± 1.58^∗∗∗^	7.09 ± 0.85	6.56 ± 0.55	6.44 ± 1.21
Jiangxi soil	${\mathrm{NH}}_{4}^{+}$-N	CK	29.2 ± 0.71	28.9 ± 0.51	26.1 ± 0.76	25.1 ± 0.41	23.4 ± 0.24
		DZ	28.8 ± 1.22	39.2 ± 1.01^∗∗∗^	42.3 ± 2.51^∗∗∗^	43.7 ± 1.37^∗∗∗^	43.4 ± 2.78^∗∗∗^
	${\mathrm{NO}}_{3}^{–}$-N	CK	22.4 ± 1.27	23.2 ± 0.61	24.5 ± 1.03	26.9 ± 0.72	31.6 ± 0.28
		DZ	23.8 ± 1.09	20.2 ± 0.35^∗^	18.1 ± 0.03^∗∗∗^	18.0 ± 0.29^∗∗∗^	19.9 ± 0.15^∗∗∗^
	MBN	CK	36.4 ± 2.45	37.2 ± 0.54	32.9 ± 1.07	32.4 ± 0.54	29.9 ± 0.69
		DZ	35.9 ± 3.12	9.45 ± 1.51^∗∗∗^	12.3 ± 0.56^∗∗∗^	24.7 ± 1.56	27.6 ± 0.23
	DAA	CK	3.93 ± 0.34	2.23 ± 0.47	3.32 ± 0.56	3.61 ± 0.65	4.21 ± 1.64
		DZ	5.01 ± 0.87	29.8 ± 1.38^∗∗∗^	11.0 ± 0.45^∗∗^	4.76 ± 0.75	6.54 ± 1.81

*The data presented are the means and standard deviations of three separate experiments. ${\mathrm{NH}}_{4}^{+}$-N (mg kg^-*1*^ soil), ${\mathrm{NO}}_{3}^{–}$-N (mg kg^-*1*^ soil), MBN (microbial biomass nitrogen, mg kg^-*1*^ soil), DAA (dissolved Amino Acids, mg kg^-*1*^ soil). The levels of statistical significance relative to the control are represented by asterisks (^∗^p < 0.05, ^∗∗^p < 0.01, ^∗∗∗^p < 0.001)*.

### Changes in Abundance of 16S rRNA and N-Cycling Functional Marker Genes

**FIGURE 2:**
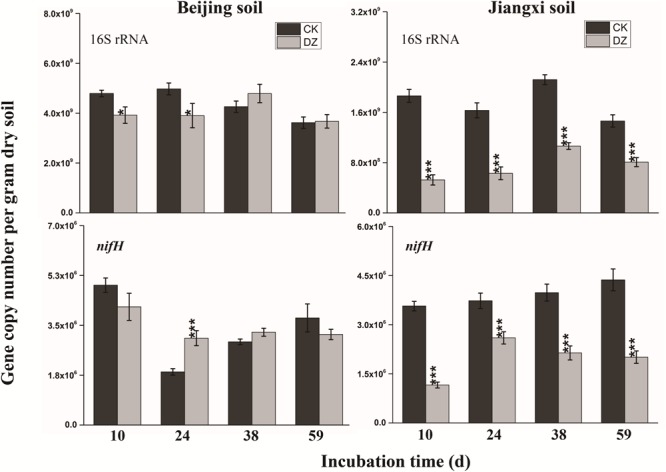
Gene copy number per gram dry soil over time for the 16S rRNA gene and nitrogen-fixing gene (*nifH*) present in two types of soil fumigated with Dazomet (DZ) or unfumigated (CK). Error bars represent standard errors of the means (*n* = 3). Statistically significant differences between the control and fumigation treatments at a specific time points are shown by asterisks according to the level of significance (^∗^*p* < 0.05, ^∗∗^*p* < 0.01, ^∗∗∗^*p* < 0.001).

**FIGURE 3:**
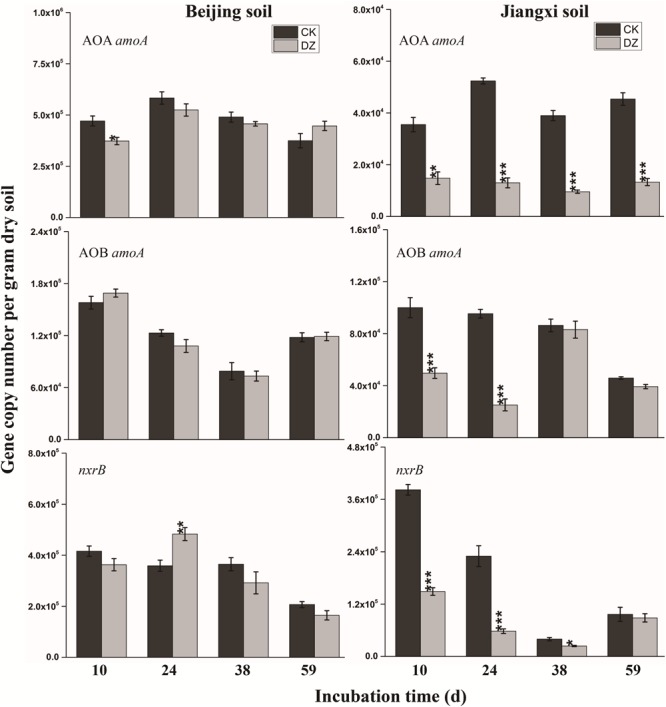
Gene copy number per gram dry soil over time for three key genes (AOA *amoA*, AOB *amoA, nxrB*) involved in nitrification in two types of soil fumigated with Dazomet (DZ) or unfumigated (CK). Error bars represent standard errors of the means (*n* = 3). Statistically significant differences between the control and fumigation treatments at a specific time point are shown by asterisks (^∗^*p* < 0.05, ^∗∗^*p* < 0.01, ^∗∗∗^*p* < 0.001).

**FIGURE 4:**
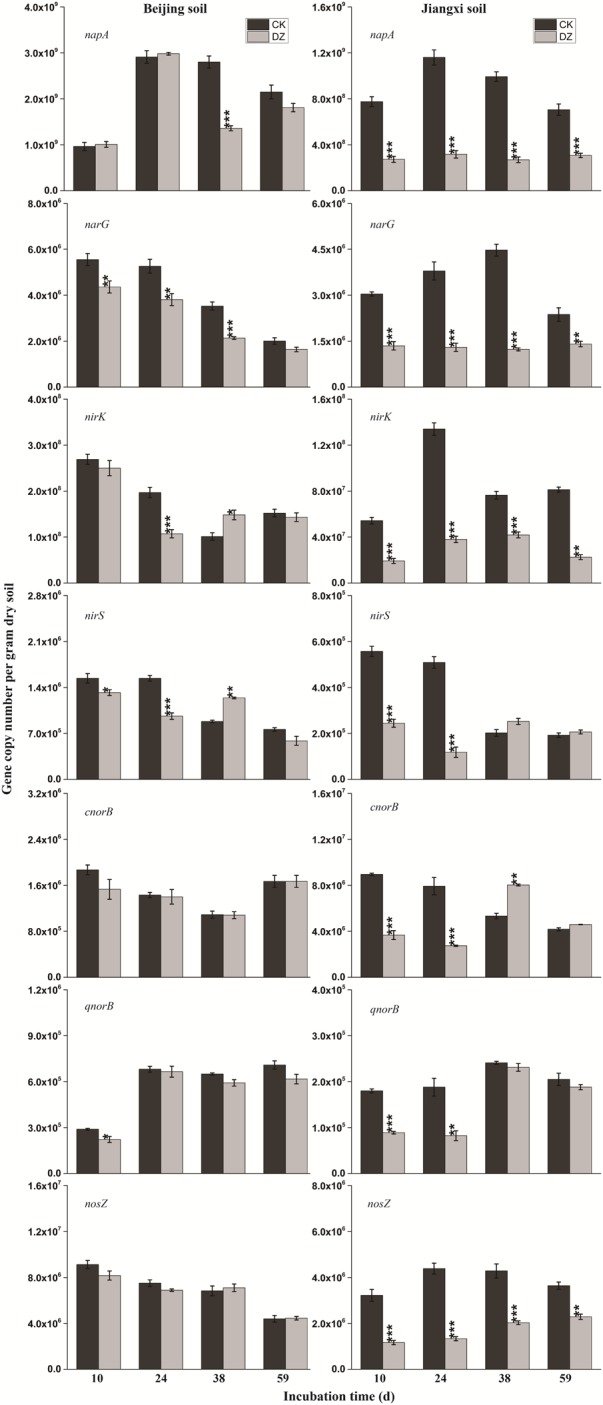
Gene copy number per gram dry soil over time for the seven key genes (*napA, narG, nirK, nirS, cnorB, qnorB*, and *nosZ*) involved in bacterial denitrification process when present in two types of soil fumigated with Dazomet (DZ) or unfumigated (CK). Error bars represent the standard errors of the means (*n* = 3). Statistically significant differences between the control and fumigant treatments at a specific time point are shown by asterisks (^∗^*p* < 0.05, ^∗∗^*p* < 0.01, ^∗∗∗^*p* < 0.001).

Statistical analysis showed that DZ significantly decreased 16S rRNA gene copy number, but this inhibitory effect was most evident in fumigated Jiangxi soil than fumigated Beijing soil (Figure 2). In addition, total bacterial abundance following DZ fumigation of Beijing soil recovered to the control level 38 days after treatment. In contrast, DZ fumigation of Jiangxi soil inhibited total bacterial abundance for the entire incubation period. The copy number of the observed 11 N-cycling functional genes (*nifH*, AOA *amoA*, AOB *amoA, nxrB, napA, narG, nirK, nirS, cnorB, qnorB*, and *nosZ*) is summarized in Figures 2–4.

When the changes in gene abundance were examined by soil type, DZ fumigation of Beijing produced only a transient decrease in the abundance of the genes AOA *amoA, napA, narG, nirK, nirS*, and *qnorB.* Their abundance recovered to the control levels 38 days after fumigation. During this period, the genes AOB *amoA, cnorB*, and *nosZ* were relatively stable. DZ fumigation of Jiangxi soil, however, resulted in the long-term (observed at 59 days) reduction in the abundance of genes *nifH*, AOA *amoA, napA, narG, nirK*, and *nosZ;* and fumigation triggered a short-term (∼24 days) depression in the abundance of genes AOB *amoA, cnorB, qnorB*, and *nirS.* However, the impact of DZ was not always inhibitory. The abundance of genes *nxrB* (*p* = 0.007), *nirK* (*p* = 0.02), *nirS* (*p* = 0.001), and *cnorB* (*p* = 0.01) on days 24 or 38 following DZ fumigation were significantly increased by fumigation, which suggested that DZ also has the ability to promote the growth of such N-cycling microorganisms.

### Changes in Soil Bacterial Diversity and Community Composition

The ‘Shannon,’ ‘ACE,’ and ‘Chao1’ Diversity Indices increased significantly (*p* < 0.01) in Beijing soil fumigated with DZ and sampled 24 days after fumigation (Table 3) relative to those in the control group, but the Simpson Diversity Index was relatively stable. This suggested that DZ can increase the diversity of soil microbial communities, but this increase appeared to be temporary as it was eliminated by day 38.

**Table 3:** Diversity indices and estimated sample coverage of Beijing soil samples at the 97% sequence identity level.

Time	Treatments	Shannon	Simpson	ACE	Chao1
Day 10	CK	6.62 ± 0.07	0.0035 ± 0.0003	3773 ± 164	3744 ± 156
	DZ	6.73 ± 0.03	0.0029 ± 0.0001	3770 ± 27.4	3765 ± 14.1
Day 24	CK	6.58 ± 0.0007	0.0036 ± 0.0001	3453 ± 112	3474 ± 103
	DZ	6.74 ± 0.04^∗∗^	0.0030 ± 0.0001	3958 ± 29.6^∗∗^	4010 ± 63.4^∗∗^
Day 38	CK	6.81 ± 0.01	0.0025 ± 0.000007	3794 ± 41.4	3769 ± 80.0
	DZ	6.70 ± 0.03	0.0029 ± 0.0001	3837 ± 93.6	3892 ± 85.2
Day 59	CK	6.76 ± 0.03	0.0028 ± 0.0001	3885 ± 125	3863 ± 157
	DZ	6.60 ± 0.04	0.0032 ± 0.0002	3487 ± 56.0	3462 ± 54.6^∗^

*The data presented are the means and standard errors of three separate experiments. The levels of statistically significance relative to the control are represented by asterisks (^∗^p < 0.05, ^∗∗^p < 0.01, ^∗∗∗^p < 0.001).*

Fumigation with DZ produced significant changes in the relative abundance of bacterial phyla as shown by changes in the abundance of the 16S rRNA gene (Supplementary Figure S1). This suggested that fumigation can have a significant impact on the structure of bacterial communities. For example, the abundance of predominant phyla such as *Acidobacteria* and *Chloroflexi* were significantly decreased 10 or 24 days after fumigation, whereas *Actinobacteria, Firmicutes, Gemmatimonadetes, Nitrospirae, Acidobacteria*, and *Chloroflexi* phyla increased in abundance 38–59 days after fumigation. *Proteobacteria*, however, the most frequently observed phylum involved in denitrification, increased initially and then significantly decreased by days 24 and 38.

### Changes in Nitrogen Cycling Microorganisms

**FIGURE 5:**
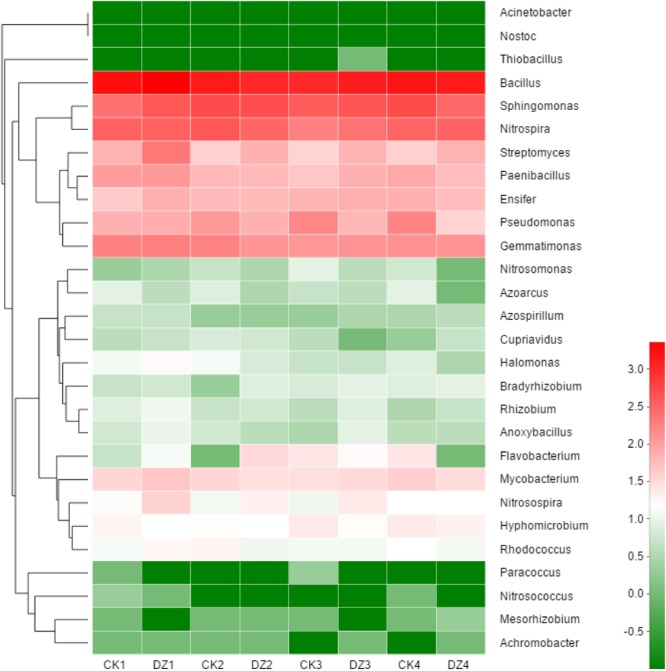
Hierarchical clustering and a heat map show changes in the relative abundance of 28 genera of bacteria involved in nitrogen cycling after fumigation with DZ. The hierarchical clustering in the figure was based on the Bray–Curtis algorithm. It shows the relative abundance of 28 genera with the highest abundance in all the samples (dark green = rare; dark red = abundant). CK: Unfumigated control; 1, 2, 3, and 4 represent the four sampling time points of 10, 24, 38, and 59 days, respectively.

All 16S rRNA gene sequences that could be assigned to known functional genes involved in nitrogen transformation were selected to assess in more detail changes to populations of N-related bacteria in Beijing soil. The 28 genera of nitrogen cycling bacteria with the greatest abundance in our trials included 7 genera of N-fixation bacteria, 5 genera of nitrification bacteria and 16 genera of denitrification bacteria (Figure 5). Populations of N_2_-fixing bacteria *Mesorhizobium, Azoarcus*, and *Paenibacillus* were initially reduced following DZ fumigation, while populations of *Bradyrhizobium* and *Rhizobium* were significantly increased by days 24 and 38. The abundance of ammonia-oxidizing bacteria *Nitrosospira* was increased initially by DZ fumigation, but *Nitrosomonas* abundance was significantly decreased by day 38. DZ fumigation caused a significant increase in the abundance of nitrite-oxidizing bacteria *Nitrospira* by day 59. DZ fumigation only initially increased denitrification bacteria *Anoxybacillus* and *Flavobacterium* but significantly increased *Streptomyces* populations for the entire incubation period of 59 days. However, many denitrification bacteria were decreased by DZ fumigation such as *Bacillus, Pseudomonas, Paracoccus, Cupriavidus, Sphingomonas*, and *Pseudomonas.* N_2_-fixing bacteria *Azospirillum* and *Nostoc*, nitrifier bacteria *Nitrosococcus* and *Nitrolancea*, denitrifier bacteria *Ensifer, Mycobacterium, Thiobacillus*, and *Rhodococcus* all maintained relatively stable populations in the fumigated Beijing soil.

### Linking N_2_O Emission to Environmental Factors and Nitrogen Cycling Bacteria

**FIGURE 6:**
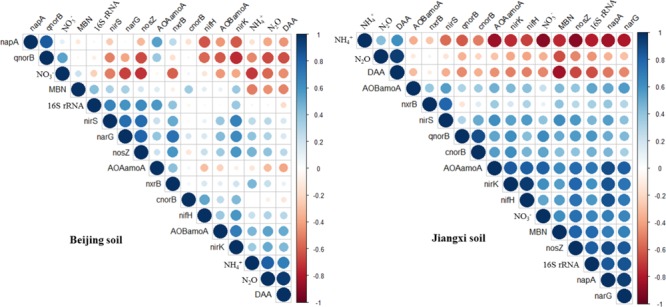
Correlation matrices between N_2_O emission rate, related functional genes and physicochemical parameters in dazomet-fumigated Beijing soil **(left)** and Jiangxi soil **(right)**. Smaller circles represent weaker Spearman’s rank correlation coefficients than larger circles.

The relationships between N_2_O emissions rate, physicochemical parameters and microbial functional genes were investigated using Spearman’s rank correlation coefficient (Figure 6). Both in fumigated Beijing soil and Jiangxi soil, the N_2_O emission rates were positively correlated with ${\mathrm{NH}}_{4}^{+}$ concentration (correlation coefficient *r* > 0.43, *p* < 0.03) and DAA (*r* > 0.94, *p* < 0.00001), but negatively correlated with MBN concentration (*r* < -0.46, *p* < 0.02). N_2_O emission rates were also significantly negatively correlated with ${\mathrm{NO}}_{3}^{–}$ concentration in Beijing soil (*r* = -0.57, *p* = 0.003) but only weakly negatively correlated in Jiangxi soil (*r* = -0.35, *p* = 0.08).

However, N_2_O emission rates in fumigated Beijing and Jiangxi soils showed no significant or consistent correlation with the abundance of microbial functional genes (Figure 6). Among the 11 genes, for example, only *qnorB* (*r* = -0.65, *p* = 0.0006) was negatively correlated with N_2_O emission rate in DZ-fumigated Beijing soil, while in fumigated Jiangxi soil the negative correlation was weak (*r* = -0.34, *p* = 0.1). Furthermore, N_2_O emission rates were positively correlated with *nosZ* (*r* = 0.41, *p* = 0.04) in DZ-fumigated Beijing soil, but negatively correlated (*r* = -0.48, *p* = 0.01) in DZ-fumigated Jiangxi soil.

## Discussion

### Effects of DZ Fumigation on Bacteria Involved in the Transformation of Nitrogen

Microbial nitrogen-fixation is a primary source of new nitrogen for terrestrial ecosystems, but many environmental parameters and farm operations such as fertilization and fumigation can moderate this biological process (Reed et al., 2010; Zheng et al., 2016). Previous research reported that fungicides (such as myclobutanil) and fumigants (such as metam sodium) can briefly inhibit the expression of the nitrogen-fixing functional gene *nifH* (Ju et al., 2016; Li et al., 2017a). We observed, however, that the abundance of *nifH* was increased in fumigated Beijing soil, suggesting that DZ can increase populations of nitrogen-fixing bacteria that contain *nifH*-encoding enzymes. Correlation analysis (Supplementary Figure S2) also showed that the abundance of *nifH* was positively correlated with the nitrogen-fixing genera *Paenibacillus* (*r* = 0.49, *p* = 0.016).

In general, the abundance of soil microorganisms is limited by nitrogen availability (Fisk and Fahey, 2001). The addition of nutrients to the soil increases the availability of nitrogen for microbes, which increases their populations (Fisk and Fahey, 2001; Fierer et al., 2003). Microorganisms are reported to adjust their efficiency of their use of carbon and nitrogen according to the environmental conditions (Mooshammer et al., 2014). Efficient use of carbon promotes microorganism abundance (Manzoni et al., 2012). The increase in the abundance of *nifH*–type bacteria 24 to 38 days after DZ fumigation in our trials might be due to the increased availability of nutrients such as ${\mathrm{NH}}_{4}^{+}$-N, compared to unfumigated soils.

Mineral nitrogen content in soil can also increase as a result of the decomposition of microbes killed by fumigation. Microbial debris has been reported to increase nitrogen (Shen et al., 1984; De Neve et al., 2004). Our research showed there was a significant increase in ${\mathrm{NH}}_{4}^{+}$-N concentration following DZ fumigation (Table 2). However, multiple ${\mathrm{NH}}_{4}^{+}$-N consumption and production pathways, including ammonia oxidation, denitrification, and dissimilatory/assimilatory nitrate reduction to ammonium, can change the ${\mathrm{NH}}_{4}^{+}$-N concentration in the soil. Our research suggested that a decrease in ammonia-oxidizing bacterial abundance might reduce ammonia oxidation, which in turn would prevent the utilization of ${\mathrm{NH}}_{4}^{+}$. Although the abundance of the AOB *amoA* functional marker gene was relatively stable in fumigated Beijing soil over the entire incubation period of 59 days, the population of *Nitrosospira* was significantly decreased by DZ fumigation during this period (Figure 5). The inhibition of AOA and AOB functional marker genes (Figure 3) resulted in a larger concentration and a prolonged presence of ${\mathrm{NH}}_{4}^{+}$-N in fumigated Jiangxi soil rather than in Beijing soil (Table 2). Microbial decomposition leads to increased microbial debris in the soil (Shen et al., 1984; De Neve et al., 2004; Yan et al., 2013, 2017). Therefore, our observation of decreased MBN and increased DAA concentrations in fumigated soil (Table 2) were most likely due to microorganism decomposition, suggesting that DZ promoted organic decomposition.

The decrease in ${\mathrm{NO}}_{3}^{–}$-N (Table 2) that we observed in fumigated soils could be attributed to microbes transforming nitrate, as reported previously (Sanders et al., 2013). The gene *nxrB* encodes nitrite oxidoreductase which converts ${\mathrm{NO}}_{2}^{–}$ to ${\mathrm{NO}}_{3}^{–}$; and *napA/narG* encodes nitrate reductase which converts ${\mathrm{NO}}_{3}^{–}$ to ${\mathrm{NO}}_{2}^{–}$ (Canfield et al., 2010). We observed that the abundance of *nxrB* and *napA/narG* were significantly decreased while ${\mathrm{NO}}_{3}^{–}$-N was continuously utilized in DZ-fumigated Jiangxi soil, which disassociated nitrifier/denitrifier bacterial abundance with associated nitrogen metabolism. This is inconsistent with reports that considered the abundance of microbial genes associated with N-cycling being a good predictor of potential nitrification and denitrification rates (Petersen et al., 2012). However, researchers have also demonstrated that denitrifying bacterial abundance and diversity can be decoupled from N-recycling (Attard et al., 2011; Dandie et al., 2011). In theory, the abundance of a functional gene could be a better indicator of microbial activity than bacterial abundance and diversity (Ka et al., 1997). Although *napA* and *narG* genes were reduced in abundance, DZ fumigation increased the relative population of denitrifying bacteria with *napA*- or *narG*-encoding enzymes, such as *Streptomyces, Anoxybacillus*, and *Flavobacterium* genera (Figure 5), which are known to have a role in ${\mathrm{NO}}_{3}^{–}$ metabolism.

Dazomet fumigation caused a significant decrease in populations of *Pseudomonas, Nitrosomonas*, and *Paracoccus* genera soon after fumigation, based on a reduction in *nirS* or *nirK*-encoding enzymes observed in our research. Previous studies reported *nirS* and *nirK* were also reduced by metam sodium fumigation. Chloropicrin fumigation, conversely, generated an increase in both *nirS* and *nirK* gene abundance (Li et al., 2017b). The diverse response of functional genes to fumigants may be due to (1) differences in the sensitivity of the functional genes; and (2) variations in the environmental performance of fumigants and their interaction with the physicochemical characteristics of different soils. DZ caused a significant increase in *nirS* and *nirK* abundance in Beijing soil that we observed on day 38, which suggested that DZ has the ability to promote the growth of *nirS* and *nirK*-type bacteria. The two different types of nitrite reductases that are responsible for nitrite reduction, either a cytochrome cd1 encoded by *nirS* or a Cu-containing enzyme encoded by *nirK*, are generally reported as the principal regulators in denitrification (Kandeler et al., 2006). In our study, we found a greater inhibition of *nirK* than *nirS* following DZ fumigation, suggesting that bacterial denitrifiers that possess copper nitrite reductase were more sensitive to DZ.

Although the abundance of *cnorB* and *qnorB* functional genes was relative stable in DZ-fumigated Beijing soil, populations of denitrifying bacteria containing these genes (such as the genera *Pseudomonas, Bacillus, Sphingomonas*, and *Cupriavidus*) were significantly reduced. We also observed that the abundance of denitrifying bacteria containing these genes was also reduced in DZ-fumigated Jiangxi soil. Two different nitric oxide reductases (Nor), which are encoded by the quinol-oxidizing single-subunit class (*qnorB*) and cytochrome bc-type complex (*cnorB*), are responsible for catalyzing the reduction of NO to N_2_O (Braker and Tiedje, 2003). The poor stability of nitric oxide reductases, together with the cytotoxic effects of NO, results in the nitric oxide reduction process receiving less research attention. However, NO is unfavorable to many biological processes, such as biofilm formation, symbiosis and quorum sensing (Terasaka et al., 2017). Bacteria contain protective mechanisms to avoid the cytotoxic effects of NO during the process of denitrification. Nitric oxide reduction (NO to N_2_O) is the most direct way to consume nitric oxide in soil. The decrease in the abundance of *cnorB* and *qnorB*-type denitrifiers, which would reduce NO consumption, results in less cytotoxic NO accumulation and emissions. Furthermore, nitric oxide reduction in the denitrification process is considered to be the primary source of N_2_O produced in the soil (Ma et al., 2008). The inhibition of this process would theoretically cause the accumulation of large amounts of N_2_O. We observed a significant increase in N_2_O emissions in DZ-fumigated soil than unfumigated treatments (Figure 1). However, the abundance of denitrifying bacteria containing *nosZ*, such as the predominant genera *Pseudomonas*, as well as *Paracoccus, Cupriavidus*, and *Sphingomonas*, decreased following DZ fumigation, while at the same time we observed an increase in the abundance of *Streptomyces*. Multiple pathways are known to lead to N_2_O production including nitrification, nitrifier-denitrification, heterotrophic nitrification and chemical denitrification. Further research is needed to fully understand the pathways of N_2_O production or reduction by denitrifying bacteria in fumigated soil.

### Effects of DZ Fumigation on N_2_O Emissions

Increased N_2_O production in soil has been attributed to the use of one or more pathways used by microbes involved in the nitrogen transformation processes (Ravishankara et al., 2009). We observed a significant correlation between N_2_O emissions and increased concentrations of ${\mathrm{NH}}_{4}^{+}$ and DAA. We surmise that the observed increase in N_2_O emissions from fumigated soil was due the availability of alternative electron acceptors and donors that became available due to significant increases in ${\mathrm{NH}}_{4}^{+}$-N and DAA. Conversely, the significant reduction in N_2_O emissions after fumigation might be due to insufficient ${\mathrm{NH}}_{4}^{+}$-N and DAA. Yan et al. (2015) observed similar correlations between increased N_2_O emissions and increased DAA concentrations. As ${\mathrm{NH}}_{4}^{+}$-N and DAA concentrations decreased, N_2_O emissions reduced because electron donors and acceptors for microbial N_2_O formation become scarce, which then limits N_2_O emissions (Harter et al., 2014).

Previous studies identified ammonium as the main source for N_2_O emissions, second only to N-based fertilizers (Bremner and Blackmer, 1979). Other research suggested that substrate availability (such as dissolved organic carbon, ${\mathrm{NO}}_{3}^{–}$ and ${\mathrm{NH}}_{4}^{+}$) was a major factor limiting N_2_O emissions in dry ecosystems (Liu et al., 2013). Assimilation of nitrogen from an exogenous source, such as dissolved organic nitrogen and inorganic nitrogen, was required for organism’s growth and biosynthesis (Sanders et al., 2013). Soil amended with a source of carbon significantly stimulated nitrate reduction and denitrification activity (Henry et al., 2008; Miller et al., 2008), which increased both production and consumption of N_2_O, respectively.

Production of N_2_O can be mediated by microbial nitrification and denitrification functional gene groups. However, in many cases the abundance of these nitrification and denitrification genes may not be related to the N_2_O emissions (Čuhel et al., 2010). Our research showed that the abundance of most nitrogen transformational genes (e.g., *nifH*, AOA *amoA*, AOB *amoA, nxrB* and *napA, narG, nirS, nirK, cnorB*) was not correlated with N_2_O emissions, while *qnorB* and *nosZ* genes showed an inconsistent and unstable correlation with N_2_O emissions in Beijing and Jiangxi soils (Figure 6). This suggested that the presence of these genes might not be the main factor governing N_2_O emissions. The microbial community involved in nitrogen transformation is diverse, consisting of archaea, bacteria and fungi. Previous research reported a functional redundancy in this community that in effect decouples the relationship between functional gene abundance and N_2_O emissions (Wertz et al., 2007; Liu et al., 2013). In addition, changes in the rates of denitrification are a complex mix of the physiological activity of individual cells, denitrifier bacterial abundance and community microbe composition (Attard et al., 2011). Under specific environmental conditions, different strains of denitrifiers within a species were reported to generate different levels of denitrification and therefore community composition can influence community activity (Ka et al., 1997; Salles et al., 2009). Therefore, given this understanding based on previous research, it is reasonable to assume that functional gene abundance we observed might not necessarily be correlated with N_2_O emissions in DZ-fumigated soil. On the other hand, our results showed that soil environmental factors (${\mathrm{NH}}_{4}^{+}$, DAA, MBN) were more significantly correlated to N_2_O emissions than functional gene abundance (Figure 6), indicating that fumigant-induced shifts in nutrient availability and specific soil environmental factors have a significant impact on N_2_O emissions. Our results agree with previous research reports of N_2_O emission being more related to changes in soil environmental conditions than denitrifier microbial abundance (Attard et al., 2011; Liu et al., 2013). At the same time, other research reported that under conditions of limited carbon supply in soils, N_2_O emissions were more closely related microbial respiration than to denitrifier abundance (Henry et al., 2008; Miller et al., 2008).

### The Effect of Soil Physicochemical Parameters on N_2_O Emission Rates

The different rates of N_2_O emissions in Beijing and Jiangxi soil types (Figure 1) suggest that the soil’s physicochemical parameters are important factors governing the rates of N_2_O emissions from fumigated soil. Soil pH is one of the most important factors influencing both denitrification and N_2_O production. In general, both denitrification rate and N_2_O production increase with increasing pH values (up to the optimum pH) (Šimek and Cooper, 2002). However, higher N_2_O emissions were observed in Jiangxi (acidic soil, pH = 4.3) than in Beijing (alkaline soil, pH = 7.2) following DZ fumigation (Figure 1). We deduce that fumigant-induced changes in N_2_O production and reduction were more significant than could be attributed soil pH alone. The dissimilar physicochemical properties of the soils resulted in different responses when they were fumigated with DZ. We observed significantly higher ${\mathrm{NH}}_{4}^{+}$ and lower ${\mathrm{NO}}_{3}^{–}$ concentrations in Beijing soil following DZ fumigation compared to the control on day 10 or day 24, but continuously higher ${\mathrm{NH}}_{4}^{+}$ and lower ${\mathrm{NO}}_{3}^{–}$ in the Jiangxi soil throughout the entire incubation period of 59 days. This indicated that DZ-fumigated Jiangxi soil retained ${\mathrm{NH}}_{4}^{+}$ at higher concentrations and ${\mathrm{NO}}_{3}^{–}$ at lower concentrations for a longer time than DZ-fumigated Beijing soil.

Considering the role of functional genes, we observed a greater reduction in Jiangxi soil than in Beijing soil in the abundance of the 11 nitrogen transfer genes and the 16S rRNA gene after DZ fumigation. Researchers reported that microbes in acidic soil were more sensitive to hexaconazole fungicide than those in alkaline soil (Ju et al., 2017). We observed a temporary inhibition of the functional genes in the Beijing soil and a fast recovery to levels similar to the control, probably because Beijing soils are alkaline and DZ is less persistent in such soils. The recovery of the functional genes is therefore closely linked with the persistence of DZ in the soil. Several studies reported that pesticides such as fosthiazate and hexaconazole were more persistent in acidic soils (Singh, 2002; Pantelelis et al., 2006). Furthermore, fumigants had a faster degradation rate in sandy loam soil compared to other soil types (Qin et al., 2016), which would account for the longer persistence of DZ in Jiangxi than in Beijing soil. The longer persistence of DZ or its metabolite acted to delay the recovery of the functional genes and the gene-encoded microbes in the Jiangxi soil. We deduce that microbes inhibited initially by DZ fumigation were able to recover faster in the alkaline Beijing soil than in the more acidic Jiangxi soil where DZ concentrations were able to persist for a longer period of time.

## Conclusion

In summary, fumigation with DZ produced a significant decrease in the abundance of 16S rRNA and eleven functional genes present in microbes involved in N-cycling in a lateritic red soil (Jiangxi soil). This inhibition effect was also present in DZ-fumigated fluvo-aquic soil (Beijing soil) but weaker. DZ also temporarily stimulated bacterial diversity as well as caused a significant change in bacterial community composition. The N_2_O emissions in fumigated soil were significantly correlated with key soil environmental factors (${\mathrm{NH}}_{4}^{+}$, DAA, MBN) and not functional gene abundance. However, when the concentrations of the fumigant declined and the inhibitory effects of DZ fumigation disappeared, the soil microbial community recovered to population levels observed in unfumigated soils. The microbial recovery rate, however, depended on the physicochemical properties of the soil. Laboratory research over a period of 3 months that examined changes in bacterial RNA levels can provides a useful and reliable method for examining the response of N-cycling microbes to soil fumigation with DZ. However, one needs to take into account that under non-destructive sampling how the nitrogen cycling microorganism response to DZ fumigation. In addition, field work using similar methods to monitor changes populations of N-cycling bacteria could usefully validate this laboratory work, particularly as it would take into account the natural microbial variability present in the soil prior to fumigation. However, our laboratory work that documents changes in the relative abundance of nitrogen transforming microbes provides (i) new mechanistic insights into the effect of soil fumigation on the structure and functioning of the N-cycling soil microbial community; and (ii) an explanation for the observed increase in N_2_O emissions as a result of soil fumigation.

## Author Contributions

WF and AC designed the study and wrote the experimental protocol. WF, BH, and XLW measured the N_2_O emissions. WF, XNW, JL, and DY performed most of the experiments. WF, XL, YL, and CO carried out the literature search and analyses. WF and QW analyzed the data. WF, QW, and AC were responsible for the overall design and wrote the scientific paper.

## Conflict of Interest Statement

The authors declare that the research was conducted in the absence of any commercial or financial relationships that could be construed as a potential conflict of interest.

## Footnotes

1 http://drive5.com/uparse/

2 http://rdp.cme.msu.edu/

3 https://www.ncbi.nlm.nih.gov/
