# Supplementary material for: Responses of Nitrogen-Cycling Microorganisms to Dazomet Fumigation
Source: Front Microbiol. 2018 Oct 23;9:2529. doi: 10.3389/fmicb.2018.02529 (PMC6206233; doi:10.3389/fmicb.2018.02529)
Supplement: Supplementary file 1 [file Table_1.docx]

**Supplementary Information**

**Responses of nitrogen-cycling microorganisms to dazomet fumigation**

Wensheng Fang, Dongdong Yan, Xianli Wang, Bin Huang, Xiaoning Wang, Jie Liu, Xiaoman Liu, Yuan Li, Canbin Ouyang, Qiuxia Wang*, Aocheng Cao*

**Table S1** Primers used for quantitative PCR

**Table S2** Quantitative PCR reaction mixtures and thermal profiles for different target genes.

**Table S3** qPCR parameters (efficiency, slope and R^2^) for evaluation of the each target gene assay

**Table S4** “Most concerned” denitrification and N fixing bacteria

**Table S5** The collected nitrogen cycle related functional microbes

**Figure S1** Relative abundance of the 16S rRNA gene sequences in bacterial phyla present in soil fumigated with dazomet (DZ). Different colors were used to represent the top 13 phyla whose relative abundance was greater than 1%. CK: Unfumigated control; 1, 2, 3, and 4 represent the four sampling time points of 10, 24, 38, and 59 days, respectively.

**Figure S2** Hierarchical clustering of genera of bacteria involved in nitrogen cycling and a heat map showing the correlation matrices, related functional genes and physicochemical parameters after Beijing soil is fumigated with dazomet. The top hierarchical clustering in the figure was performed based on the Bray-Curtis algorithm, and the left heat map was constructed based on the 28 genera of nitrogen cycling bacteria in all the samples. The legend with different color intensities are used to represent the normalized relative size of the correlation in each row, based on Spearman’s rank correlation coefficient (*p<0.05, **<0.01, ***<0.001).

Table S1 Primers used for quantitative PCR

| **Gene** | **Primer** | **Sequence** | **[bp]** | **Reference** |
| --- | --- | --- | --- | --- |
| *16S*  *rRNA* | 341f  797r | CCTACGGGAGGCAGCAG  GGACTACCAGGGTATCTAATCCTGTT | 466 | [Muyzer et al (1993](#_ENREF_6))  [Nadkarni et al (2002](#_ENREF_7)) |
| *nifH* | nifHF  nifHR | AAAGGYGGWATCGGYAARTCCACCAC  TTGTTSGCSGCRTACATSGCCATCAT | 458 | [Rosch et al (2002](#_ENREF_10)) |
| archaeal  *amoA* | amo19F  CrenamoA616r48x | ATGGTCTGGCTWAGACG  GCCATCCABCKRTANGTCCA | 624 | [Leininger et al (2006](#_ENREF_4))  [Schauss et al (2009](#_ENREF_12)) |
| bacterial  *amoA* | amoA1F  amoA2R | GGGGTTTCTACTGGTGGT  CCCCTCKGSAAAGCCTTCTTC | 500 | [Rotthauwe et al (1997](#_ENREF_11)) |
| *nirS* | cd3af  R3cd | GTNAAYGTNAARGARACNGG  GASTTCGGRTGSGTCTTGA | 413 | [Michotey et al (2000](#_ENREF_5))  [Throback et al (2004](#_ENREF_13)) |
| *napA* | napA4f  napA4r | TGGACVATGGGYTTYAAYC  ACYTCRCGHGCVGTRCCRCA | 152 | Bru et al 2007 |
| *qnorB* | qnorB2F  qnorB5R | GGNCAYCARGGNTAYGA  ACCCANAGRTGNACNACCCACCA | 262 | Gesche et al 2003 |
| *cnorB* | cnorB2F | GACAAGNNNTACTGGTGGT | 389 | Gesche et al 2003 |
|  | cnorB6R | GAANCCCCANACNCCNGC |  |  |
| *narG* | narG2F | TAYGTSGGGCAGGARAAACTG | 110 | López-Gutiérrez et al 2004 |
|  | narG2R | CGTAGAAGAAGCTGGTGCTGTT |  |  |
| *nosZ* | nosZ2F  nosZ2R | CGCRACGGCAASAAGGTSMSSGT  CAKRTGCAKSGCRTGGCAGAA | 267 | [Henry et al (2006](#_ENREF_3)) |
| *nirK* | *nirK*F1aCu  *nirK*R3Cu | ATCATGGTSCTGCCGCG  GCCTCGATCAGRTTGTGGTT | 473 | Henry et al. (2004) |
| *nxrB* | nxrB169f  nxrB638r | TAC ATG TGG TGG AACA  CGG TTC TGG TCR ATC A | 485 | Pester M, et al (2014) |

Table S2 Quantitative PCR reaction mixtures and thermal profiles for different target genes.

| **Target gene** | **Reaction mixture** | **Volumes (ul)** | **Thermal profile** | **Reference** |
| --- | --- | --- | --- | --- |
| *16S rRNA gene* | SsoFast EvaGreen® Supermix  341f  797R  Template  PCR water | 10  1  1  1  7 | 53^o^C-45s  72^o^C-45s  44 cycles | Modified after Harter *et al.* (2014) |
| *nifH* | SsoFast EvaGreen® Supermix  nifHF  nifHR  Template  PCR water | 10  1  1  1  7 | 55^o^C-45s  72^o^C-45s  44 cycles | Modified after Harter *et al.* (2014) |
| *archaeal amoA* | SsoFast EvaGreen® Supermix  Amo19F  CrenamoA616r48x  Template  PCR water | 10  1  1  1  7 | 53^o^C-45s  72^o^C-45s  44 cycles | Modified after Harter *et al.* (2014) |
| *bacterial*  *amoA* | SsoFast EvaGreen® Supermix  AmoA1F  AmoA2R  Template  PCR water | 10  1  1  1  7 | 55^o^C-45s  72^o^C-45s  44 cycles | Modified after Harter *et al.* (2014) |
| *nirS* | SsoFast EvaGreen® Supermix  Cd3af  R3cd  Template  PCR water | 10  1  1  1  7 | 57^o^C-45s  72^o^C-45s  44 cycles | Modified after Harter *et al.* (2014) |
| *napA* | SsoFast EvaGreen® Supermix  NapA4f  NapA4r  Template  PCR water | 10  1  1  1  7 | 55^o^C-30s  72^o^C-30s  44 cycles | Modified after Bru *et al.* (2007) |
| *qnorB* | SsoFast EvaGreen® Supermix  QnorB2F  QnorB5R  Template  PCR water | 10  1  1  1  7 | 55^o^C-35s  72^o^C-45s  44 cycles | Modified after Gesche *et al.* (2003) |
| *cnorB* | SsoFast EvaGreen® Supermix  CnorB2F  CnorB6R  Template  PCR water | 10  1  1  1  7 | 55^o^C-35s  72^o^C-45s  44 cycles | Modified after Gesche *et al.* (2003) |
| *narG* | SsoFast EvaGreen® Supermix  NarG2F  NarG2R  Template  PCR water | 10  1  1  1  7 | 55^o^C-30s  72^o^C-45s  44 cycles | Modified after López-Gutiérrez *et al.* (2004) |
| *nosZ* | SsoFast EvaGreen® Supermix  NosZ2F  NosZ2R  Template  PCR water | 10  1  1  1  7 | 60^o^C-35s  72^o^C-30s  44 cycles | Modified after Harter *et al.* (2014) |
| *nirK* | SsoFast EvaGreen® Supermix  NirKF1aCu  NirKR3Cu  Template  PCR water | 10  1  1  1  7 | 60^o^C-35s  72^o^C-30s  44 cycles | Modified after Harter *et al.* (2004) |
| *nxrB* | SsoFast EvaGreen® Supermix  nxrB169f  nxrB638r  Template  PCR water | 10  1  1  1  7 | 55^o^C-45s  72^o^C-45s  44 cycles | Modified after Pester *et al.* (2014) |

Table S3 qPCR parameters (efficiency, slope and R^2^) for evaluation of the each target gene assay

| **Target gene** | **Efficiency (%)** | **Slope** | **R^2^** |
| --- | --- | --- | --- |
| *16S rRNA gene* | 102 | -3.26 | 0.998 |
| *nifH* | 105 | -3.19 | 0.994 |
| *Archaeal amoA* | 95.6 | -3.43 | 0.999 |
| *Bacterial amoA* | 104 | -3.22 | 0.999 |
| *nirS* | 97.5 | -3.38 | 0.998 |
| *napA* | 101 | -3.29 | 0.995 |
| *qnorB* | 83.6 | -3.74 | 0.998 |
| *cnorB* | 88.4 | -3.63 | 0.999 |
| *narG* | 90.8 | -3.58 | 0.999 |
| *nosZ* | 98.7 | -3.35 | 0.995 |
| *nirK* | 104 | -3.21 | 0.999 |
| *nxrB* | 92.2 | -3.48 | 0.999 |

**References**

Bru D, Sarr A, Philippot L. Relative abundances of proteobacterial membrane-bound and periplasmic nitrate reductases in selected environments. *Appl Environ Microbiol*, 2007, 73(18):5971-4.

Gesche Braker, and James M. Tiedje. "Nitric oxide reductase (norB) genes from pure cultures and environmental samples." *Appl Environ Microbiol*, 2003, 69.6: 3476-3483.

Harter J, Krause HM, Schuettler S, Ruser R, Fromme M, Scholten T, *et al..* Linking N2O emissions from biochar-amended soil to the structure and function of the N-cycling microbial community. *Isme Journal*, 2014; 8: 660.

Henry S, Baudoin E, López-Gutiérrez JC, Martin-Laurent F, Brauman A, Philippot L. Quantification of denitrifying bacteria in soils by *nirK* gene targetedreal-time PCR. *J Microbiol Meth*, 2004, 59:327–35.

Henry S, Bru D, Stres B, Hallet S, Philippot L. Quantitative detection of the nosZ gene, encoding nitrous oxide reductase, and comparison of the abundances of 16S rRNA, narG, nirK, and nosZ genes in soils. *Appl Environ Microbiol*, 2006, 72: 5181-5189

Leininger S, Urich T, Schloter M, Schwark L, Qi J, Nicol GW. Archaea predominate among ammonia-oxidizing prokaryotes in soils. *Nature*, 2006, 442: 806-809.

López-Gutiérrez J C, Henry S, Hallet S, *et al.* Quantification of a novel group of nitrate-reducing bacteria in the environment by real-time PCR. *J Microbiol Meth,* 2004, 57(3): 399-407.

Michotey V, Mejean V, Bonin P. Comparison of methods for quantification of cytochrome cd(1)-denitrifying bacteria in environmental marine samples. *Appl Environ Microbiol,* 2000*,* 66: 1564-1571

Muyzer G, Dewaal EC, Uitterlinden AG. Profiling of complex microbial populations by denaturing gradient gel electrophoresis analysis of polymerase chain reaction-amplified genes coding for 16S rRNA. *Appl Environ Microbiol*, 1993, 59: 695-700.

Nadkarni MA, Martin FE, Jacques NA, Hunter N. Determination of bacterial load by real-time PCR using a broad-range (universal) probe and primers set. *Microbiology-(UK)*, 2002, 148: 257-266.

Pester M, Maixner F, Berry D, *et al.* NxrB encoding the beta subunit of nitrite oxidoreductase as functional and phylogenetic marker for nitrite-oxidizing *Nitrospira*. *Environ Microbiol*, 2014, 16(10):3055.

Rosch C, Mergel A, Bothe H. Biodiversity of denitrifying and dinitrogen-fixing bacteria in an acid forest soil. *Appl Environ Microbiol*, 2002, 68: 3818-3829.

Rotthauwe JH, Witzel KP, Liesack W. The ammonia monooxygenase structural gene amoA as a functional marker: Molecular fine-scale analysis of natural ammonia-oxidizing populations. *Appl Environ Microbiol*, 1997, 63: 4704-4712.

Schauss K, Focks A, Leininger S, Kotzerke A, Heuer H, Thiele-Bruhn S. Dynamics and functional relevance of ammonia-oxidizing archaea in two agricultural soils. *Environ Microbiol*, 2009, 11: 446-456.

Throback IN, Enwall K, Jarvis A, Hallin S. Reassessing PCR primers targeting nirS, nirK and nosZ genes for community surveys of denitrifying bacteria with DGGE. *FEMS Microbiol Ecol*, 2004, 49: 401-417.

Table S4 “Most concerned” denitrification and N fixing bacteria

| Genus | Enzyme/Functional gene | Reference |
| --- | --- | --- |
| Gemmatimonas | nitrous oxide reductases (nosZ) | (Park et al., 2017) |
| Cupriavidus | possess the heterotrophic nitrification and aerobic denitrification ability | (Sun et al., 2016) |
| Anoxybacillus | possess the heterotrophic nitrification and aerobic denitrification ability | (Chen et al., 2015) |
| Acinetobacter | possess the heterotrophic nitrification-aerobic denitrification ability | (Zhang et al., 2012) |
| Sphingomonas | nitrite and nitric oxide reductases. | (Cua and Stein, 2014) |
| Paenibacillus | Nitrogenase(nifH) | (Wang et al., 2013) |
| Rhodococcus | possess the heterotrophic nitrification-aerobic denitrification ability | (Chen et al., 2012) |
| Thermus thermophilus | possess aerobic denitrification ability | (Alvarez et al., 2011) |
| Pseudomonas | possess aerobic denitrification ability | (Schaefer, 2009) |
| Escherichia | possess aerobic denitrification ability | (Schaefer, 2009) |
| Lactobacillus | Nitrate reductase (narGHJI) | (Brooijmans et al., 2009) |
| Neisseria | nitric oxide reductase (NorB) and nitrite reductase (AniA or NirK) | (Barth et al., 2009) |
| Agrobacetrium | nitric oxide reductase (NorB) and nitrite reductase (NirK) | (Bergaust et al., 2008) |
| Nitrosospira | nitric oxide reductase (NorB) and nitrite reductase (NirK) | (Garbeva et al., 2007) |
| Brucella | possess denitrification ability (nar, nirK, norB, nosZ) | (Haine et al., 2006) |
| Nitrosomonas | nitrite reductase (NirK) | (Beaumont et al., 2004) |
| Azoarcus | Nitrogenase(nifH) | (Hurek and Reinhold-Hurek, 2003) |
| Pyrobaculum | NO reductase | (De et al., 2003) |
| Pseudomonas | N2O reductase (nosRZDFYL) | (Arai et al., 2003) |
| Paracoccus | N2O reductase | (Hoglen and Hollocher, 1989) |
| Achromobacter | N2O reductase | (Hulse and Averill, 1990) |
| Streptomyces | possess denitrification ability | (Kumon et al., 2002) |
| Rhodobacter | Nitrate Reductase (Nap) | (Gavira et al., 2002) |
| Paracoccus | Nitrate Reductase (Nap) | (Roldán et al., 1998) |
| Haemophilus | Nitrate Reductase (Nap) | (Brigé et al., 2001) |
| Shewanella | possess denitrification ability | (Brettar et al., 2002) |
| Haloarcula | Cu-containing dissimilatory nitrite reductase (CuNiR) | (Ichiki et al., 2001) |
| Vibrio succinogenes | nitrate or nitrite Reductase | (Bokranz et al., 1983) |
| Thiosphaera pantotropha | nitrate reductases | (Bell et al., 1990) |
| Thiobacillus | possess denitrification ability | (Claus and Kutzner, 1985) |
| Rhizobium | Nitrogenase(nifH) | (O'Hara and Daniel, 1985) |
| Ralstonia | NO reductases | (Cramm et al., 1999) |
| Nostoc | Nitrogenase | (Silvester and Smith, 1969) |
| mycobacterium | nitrate reductase (narGHJI) | (Weber et al., 2000) |
| Hyphomicrobium | Possess denitrification and nitrogen  fixation genes | (Fesefeldt et al., 1998) |
| Escherichia | nitrate reductase | (Brondijk et al., 2004) |
| Ensifer | Possess napEFDABC, nirK, norECBQD and  nosRZDFYLX denitrification genes | (Torres et al., 2014) |
| Corynebacterium | nitrate, nitrite, and nitric oxide reductase | (Renner and Becker, 1970) |
| Comamonas | Nitrate and Nitrite Reductases | (Renner and Becker, 1970) |
| Bradyrhizobium | Possess nirK, norC and nosZ denitrification genes | (Mesa et al., 2004) |
| Bacillus | possess denitrification ability | (Denariaz, 1989) |
| Azospirillum | dissimilatory nitrite reductase | (Kloos et al., 2001) |
| Alcaligenes | Nitric Oxide Reductases | (Cramm et al., 1997) |
| Achromobacter | Nitrite Reductase | (Godden et al., 1991) |

**Reference:**

Alvarez, L., Bricio, C., Gómez, M.J., Berenguer, J., 2011. Lateral transfer of the denitrification pathway genes among Thermus thermophilus strains. Applied & Environmental Microbiology 77, 1352-1358.

Arai, H., Mizutani, M., Igarashi, Y., 2003. Transcriptional regulation of the nos genes for nitrous oxide reductase in Pseudomonas aeruginosa. Microbiology 149, 29-36.

Barth, K.R., Isabella, V.M., Clark, V.L., 2009. Biochemical and genomic analysis of the denitrification pathway within the genus Neisseria. Microbiology 155, 4093-4103.

Beaumont, H.J., Lens, S.I., Reijnders, W.N., Westerhoff, H.V., van Spanning, R.J., 2004. Expression of nitrite reductase in Nitrosomonas europaea involves NsrR, a novel nitrite-sensitive transcription repressor. Molecular Microbiology 54, 148.

Bell, L.C., Richardson, D.J., Ferguson, S.J., 1990. Periplasmic and membrane-bound respiratory nitrate reductases in Thiosphaera pantotropha : The periplasmic enzyme catalyzes the first step in aerobic denitrification. Febs Letters 265, 85-87.

Bergaust, L., Shapleigh, J., Frostegård, A., Bakken, L., 2008. Transcription and activities of NOx reductases in Agrobacterium tumefaciens. Environmental Microbiology 10, 3070-3081.

Bokranz, M., Katz, J., Schröder, I., Roberton, A.M., Kröger, A., 1983. Energy metabolism and biosynthesis of Vibrio succinogenes growing with nitrate or nitrite as terminal electron acceptor. Archives of Microbiology 135, 36-41.

Brettar, I., Christen, R., Höfle, M.G., 2002. Shewanella denitrificans sp. nov., a vigorously denitrifying bacterium isolated from the oxic-anoxic interface of the Gotland Deep in the central Baltic Sea. International Journal of Systematic & Evolutionary Microbiology 52, 2211.

Brigé, A., Cole, J.A., Hagen, W.R., Guisez, Y., Van, J.B., 2001. Overproduction, purification and novel redox properties of the dihaem cytochrome c, NapB, from Haemophilus influenzae. Biochemical Journal 356, 851-858.

Brondijk, T.H., Nilavongse, A., Filenko, N., Richardson, D.J., Cole, J.A., 2004. NapGH components of the periplasmic nitrate reductase of Escherichia coli K-12: location, topology and physiological roles in quinol oxidation and redox balancing. Biochemical Journal 379, 47.

Brooijmans, R.J., de Vos, W.M., Hugenholtz, J., 2009. Lactobacillus plantarum WCFS1 electron transport chains. Applied & Environmental Microbiology 75, 3580.

Chen, J., Zheng, J., Li, Y., Hao, H.H., Chen, J.M., 2015. Characteristics of a novel thermophilic heterotrophic bacterium, Anoxybacillus contaminans HA, for nitrification-aerobic denitrification. Applied Microbiology & Biotechnology 99, 10695-10702.

Chen, P., Li, J., Li, Q.X., Wang, Y., Li, S., Ren, T., Wang, L., 2012. Simultaneous heterotrophic nitrification and aerobic denitrification by bacterium Rhodococcus sp. CPZ24. Bioresource Technology 116, 266-270.

Claus, G., Kutzner, H.J., 1985. Physiology and kinetics of autotrophic denitrification by Thiobacillus denitrificans. Applied Microbiology & Biotechnology 22, 283-288.

Cramm, R., Pohlmann, A., Friedrich, B., 1999. Purification and characterization of the single‐component nitric oxide reductase from Ralstonia eutropha H16. Febs Letters 460, 6-10.

Cramm, R., Siddiqui, R.A., Friedrich, B., 1997. Two isofunctional nitric oxide reductases in Alcaligenes eutrophus H16. Journal of Bacteriology 179, 6769-6777.

Cua, L.S., Stein, L.Y., 2014. Characterization of denitrifying activity by the alphaproteobacterium, Sphingomonas wittichii RW1. Front Microbiol 5, 404.

De, V.S., Strampraad, M.J., Lu, S., Moënne-Loccoz, P., Schröder, I., 2003. Purification and characterization of the MQH2:NO oxidoreductase from the hyperthermophilic archaeon Pyrobaculum aerophilum. Journal of Biological Chemistry 278, 35861-35868.

Denariaz, G., 1989. A halophilic denitrifier, Bacillus halodenitrificans sp. nov. Int.j.syst.bacteriol 39, 145-151.

Fesefeldt, A., Kloos, K., Bothe, H., Lemmer, H., Gliesche, C.G., 1998. Distribution of denitrification and nitrogen fixation genes in Hyphomi. Canadian Journal of Microbiology 44, 181-186.

Garbeva, P., Baggs, E.M., Prosser, J.I., 2007. Phylogeny of nitrite reductase (nirK) and nitric oxide reductase (norB) genes from Nitrosospira species isolated from soil. Fems Microbiology Letters 266, 83-89.

Gavira, M., Roldán, M.D., Castillo, F., Morenovivián, C., 2002. Regulation of nap Gene Expression and Periplasmic Nitrate Reductase Activity in the Phototrophic Bacterium Rhodobacter sphaeroides DSM158. Journal of Bacteriology 184, 1693-1702.

Godden, J.W., Turley, S., Teller, D.C., Adman, E.T., Liu, M.Y., Payne, W.J., Legall, J., 1991. The 2.3 angstrom X-ray structure of nitrite reductase from Achromobacter cycloclastes. science 253, 438-442.

Haine, V., Dozot, M., Dornand, J., Letesson, J.J., De, B.X., 2006. NnrA is required for full virulence and regulates several Brucella melitensis denitrification genes. Journal of Bacteriology 188, 1615.

Hoglen, J., Hollocher, T.C., 1989. Purification and some characteristics of nitric oxide reductase-containing vesicles from Paracoccus denitrificans. Journal of Biological Chemistry 264, 7556-7563.

Hulse, C.L., Averill, B.A., 1990. Isolation of a high specific activity pink, monomeric nitrous oxide reductase from Achromobacter cycloclastes. Biochemical & Biophysical Research Communications 166, 729-735.

Hurek, T., Reinhold-Hurek, B., 2003. Azoarcus sp strain BH72 as a model for nitrogen-fixing grass endophytes. Journal of Biotechnology 106, 169.

Ichiki, H., Tanaka, Y., Mochizuki, K., Yoshimatsu, K., Sakurai, T., Fujiwara, T., 2001. Purification, characterization, and genetic analysis of Cu-containing dissimilatory nitrite reductase from a denitrifying halophilic archaeon, Haloarcula marismortui. Journal of Bacteriology 183, 4149.

Kloos, K., Mergel, A., Rösch, C., Bothe, H., 2001. Denitrification within the genus Azospirillum and other associative bacteria. Australian Journal of Plant Physiology 28, 991-998.

Kumon, Y., Sasaki, Y., Kato, I., Takaya, N., Shoun, H., Beppu, T., 2002. Codenitrification and Denitrification Are Dual Metabolic Pathways through Which Dinitrogen Evolves from Nitrate in Streptomyces antibioticus. Journal of Bacteriology 184, 2963-2968.

Mesa, S., Jd, A., Bedmar, E., Delgado, M.J., 2004. Expression of nir, nor and nos denitrification genes from Bradyrhizobium japonicum in soybean root nodules. Physiologia Plantarum 120, 205–211.

O'Hara, G.W., Daniel, R.M., 1985. Rhizobial denitrification: a review. Soil Biology & Biochemistry 17, 1-9.

Park, D., Kim, H., Yoon, S., 2017. Nitrous Oxide Reduction by an Obligate Aerobic Bacterium, Gemmatimonas aurantiaca Strain T-27. Appl Environ Microbiol 83, e00502-00517.

Renner, E.D., Becker, G.E., 1970. Production of nitric oxide and nitrous oxide during denitrification by Corynebacterium nephridii. Journal of Bacteriology 101, 821-826.

Roldán, M.D., Sears, H.J., Cheesman, M.R., Ferguson, S.J., Thomson, A.J., Berks, B.C., Richardson, D.J., 1998. Spectroscopic characterization of a novel multiheme c-type cytochrome widely implicated in bacterial electron transport. Journal of Biological Chemistry 273, 28785-28790.

Schaefer, R., 2009. The transcription factor DNR from Pseudomonas aeruginosa specifically requires nitric oxide and haem for the activation of a target promoter in Escherichia coli. Microbiology 155, 2838-2844.

Silvester, W.B., Smith, D.R., 1969. Nitrogen Fixation by Gunnera-Nostoc Symbiosis. Nature 224, 1231-1231.

Sun, Z., Lv, Y., Liu, Y., Ren, R., 2016. Removal of nitrogen by heterotrophic nitrification-aerobic denitrification of a novel metal resistant bacterium Cupriavidus sp. S1. Bioresour Technol 220, 142-150.

Torres, M.J., Rubia, M.I., Peña, T.C.D.L., Pueyo, J.J., Bedmar, E.J., Delgado, M.J., 2014. Genetic basis for denitrification in Ensifer meliloti. BMC Microbiol. 14, 1-10.

Wang, L., Zhang, L., Liu, Z., Liu, Z., Zhao, D., Liu, X., Zhang, B., Xie, J., Hong, Y., Li, P., 2013. A minimal nitrogen fixation gene cluster from Paenibacillus sp. WLY78 enables expression of active nitrogenase in Escherichia coli. Plos Genetics 9, e1003865.

Weber, I., Fritz, C., Ruttkowski, S., Kreft, A., Bange, F.C., 2000. Anaerobic nitrate reductase (narGHJI) activity of Mycobacterium bovis BCG in vitro and its contribution to virulence in immunodeficient mice. Molecular Microbiology 35, 1017-1025.

Zhang, Q.L., Liu, Y., Ai, G.M., Miao, L.L., Zheng, H.Y., Liu, Z.P., 2012. The characteristics of a novel heterotrophic nitrification-aerobic denitrification bacterium, Bacillus methylotrophicus strain L7. Bioresource Technology 108, 35.
