# Supplementary figures and images for: Responses of Nitrogen-Cycling Microorganisms to Dazomet Fumigation
Source: Front Microbiol. 2018 Oct 23;9:2529. doi: 10.3389/fmicb.2018.02529 (PMC6206233; doi:10.3389/fmicb.2018.02529)

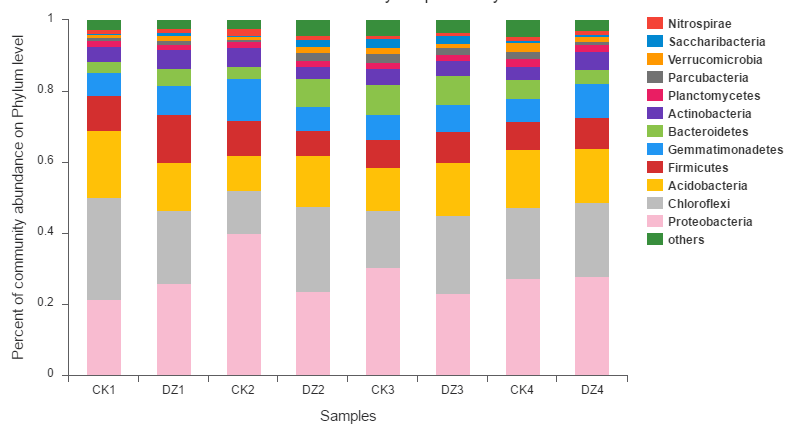

Supplement: Supplementary file 3 [file Image_1.PNG]

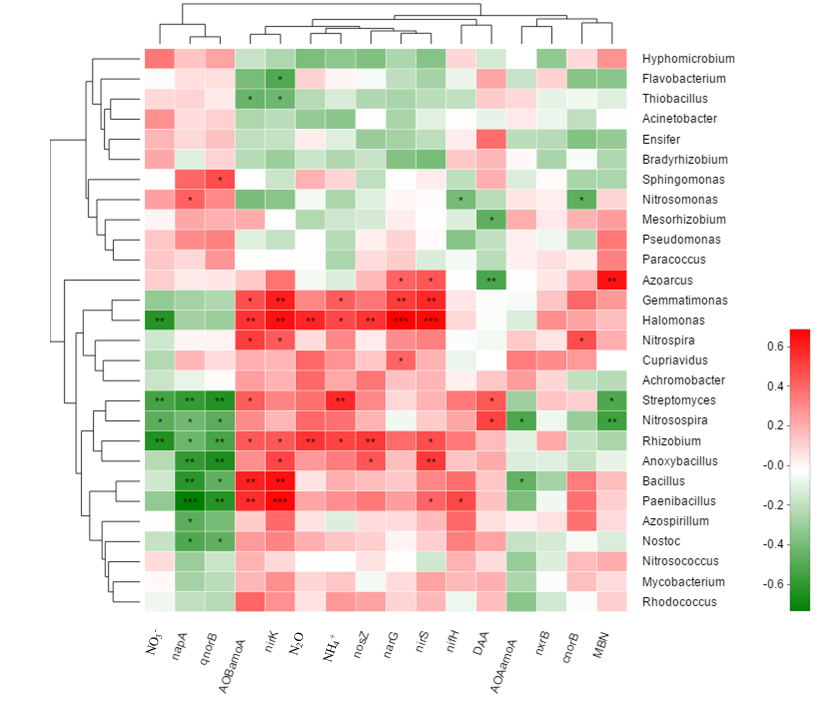

Supplement: Supplementary file 4 [file Image_2.TIF]
